# Characterization of hydromechanical stress in aerated stirred tanks up to 40 m^3^ scale by measurement of maximum stable drop size

**DOI:** 10.1186/1754-1611-8-17

**Published:** 2014-07-07

**Authors:** Andreas Daub, Marina Böhm, Stefanie Delueg, Markus Mühlmann, Gerhard Schneider, Jochen Büchs

**Affiliations:** 1 AVT.Biochemical Engineering, RWTH Aachen University, Worringerweg 1, Aachen 52074, Germany; 2 Sandoz GmbH, Anti-Infectives Operations Development, Biochemiestraße 10, Kundl A-6250, Austria

**Keywords:** Drop size, Turbulence, Hydromechanical stress, Energy dissipation, Aeration, Multiphase reactors

## Abstract

**Background:**

Turbulence intensity, or hydromechanical stress, is a parameter that influences a broad range of processes in the fields of chemical engineering and biotechnology. Fermentation processes are often characterized by high agitation and aeration intensity resulting in high gas void fractions of up to 20% in large scale reactors. Very little experimental data on hydromechanical stress for such operating conditions exists because of the problems associated with measuring hydromechanical stress under aeration and intense agitation.

**Results:**

An indirect method to quantify hydromechanical stress for aerated operating conditions by the measurement of maximum stable drop size in a break-up controlled dispersion was applied to characterize hydromechanical stress in reactor scales of 50 L, 3 m^3^ and 40 m^3^ volume with a broad range of operating conditions and impeller geometries (Rushton turbines). Results for impellers within each scale for the ratio of maximum to specific energy dissipation rate ϕ based on measured values of maximum stable drop size for aerated operating conditions are qualitatively in agreement with results from literature correlations for unaerated operating conditions. Comparison of data in the different scales shows that there is a scale effect that results in higher values for ϕ in larger reactors. This behavior is not covered by the classic theory of turbulent drop dispersion but is in good agreement with the theory of turbulence intermittency. The data for all impeller configurations and all aeration rates for the three scales can be correlated within ±20% when calculated values for ϕ based on the measured values for d_max_ are used to calculate the maximum local energy dissipation rate. A correlation of the data for all scales and all impeller configurations in the form ϕ = 2.3∙(ϕ_unaerated_)^0.34^∙(D_R_)^0.543^ is suggested that successfully models the influence of scale and impeller geometry on ϕ for aerated operating conditions.

**Conclusions:**

The results show that besides the impeller geometry, also aeration and scale strongly influence hydromechanical stress. Incorporating these effects is beneficial for a successful scale up or scale down of this parameter. This can be done by applying the suggested correlation or by measuring hydromechanical stress with the experimental method used in this study.

## Background

Turbulence intensity, or hydromechanical stress, is a parameter with an important impact on many different processes in the fields of chemical engineering and biotechnology. Since it governs the break-up of bubbles and drops in a turbulent flow field [1] it is very important in processes where interfacial area for mass transfer can become rate limiting [2]. Furthermore, turbulence intensity has been discussed for a long time to have a large influence on biotechnological processes by a direct action on the biological phase [3]. E.g. cell viability in microcarrier based cell culture processes can be correlated with turbulence intensity [4]. In submerged fungal fermentations turbulence intensity may interact with morphological behaviour of the fungus [5]. It was shown that volumetric power input in shake flasks is comparable to volumetric power input in stirred fermenters [6,7] for typical operating conditions. Nevertheless, even at comparable levels of volumetric power input pellets grow much larger in shake flask culture compared to cultures in stirred tank reactors because turbulence intensity is much lower than in stirred reactors [8-10]. There is a close interaction between morphology, broth rheology and agitation intensity in submerged fungal fermentations in stirred tank reactors that can impact process performance [11]. Production scale for fermentation processes can be as large as several hundred m^3^. The development of these large scale processes is conducted in lab or pilot scale fermenters. This poses the question how to scale down the conditions prevalent in large production scale to the development scale and vice versa. Typical parameters of interest may be mass transfer coefficient k_L_a, volumetric power input P/V_L_, impeller tip speed u_tip_, turbulence intensity in the form of maximum local energy dissipation rate ϵ_max_, circulation frequency t_c_, or a combination of these parameters like the energy dissipation to circulation function EDCF (first introduced by [12]) that is basically the ratio of ϵ_max_/t_c_. Based on an estimation of the values of these parameters in large scale, small scale operating conditions (agitation and aeration rates) can be estimated that resemble the respective values of these different parameters in large scale. Testing these operating conditions in small scale may reveal which of these parameters (if at all) is a suitable proxy for scaling up or scaling down a particular process. The work of Jüsten [11-13], e.g., showed that scale up of mycelial flocs can be correlated with hydromechanical stress and circulation frequency in the form of the energy dissipation to circulation function EDCF. The morphology of the fungus in this case depends on the break up in the impeller region that is governed by hydromechanical stress and the aggregation of mycelial flocs in regions where turbulence intensity is low. Circulation frequency is decisive for the time for aggregation and for the frequency the mycelial flocs pass the high intensity region close to the impeller where they break up.

The whole procedure of testing different parameter candidates strongly depends on the validity of the correlations used to calculate these parameters. Although turbulence intensity is often discussed to influence biological processes only few data exists on the influence of geometry, scale and aeration on this parameter. There are two circumstances that strongly complicate the intention of establishing comparable levels of turbulence intensity in large and small scale: first, in industrial practice, geometrical similarity throughout the scales is hardly found [14,15]. Therefore, the influence of geometry on turbulence intensity must be known for a successful scale up or scale-down of this parameter. Second, in aerobic fermentations the working medium is a multiphase gas-liquid dispersion which is characterized by a volumetric gas hold-up of up to 20% in production scale reactors [15]. Of course, this is accompanied by much higher gas hold-ups in the vicinity of the turbulence inducing agitators. Very little is known on the influence of such high gas hold-ups on turbulence characteristics in stirred tanks because it is very hard to measure this parameter under these conditions. Particle image velocimetry (PIV) and laser Doppler anemometry (LDA) are often used to measure maximum local energy dissipation rates in small scale, single-phase reactors [16-23]. However, these methods cannot be applied in high gas hold-up conditions. Most available data was measured in lab scale with low agitation intensity. A review on existing data for maximum local energy dissipation was presented in [24].

It would be desirable to have a simple and practical method at hand to experimentally investigate turbulence intensity in large-scale multiphase reactors. This would enable a more rational approach to the scale-up and scale-down of this parameter for existing process equipment and it would help to throw light onto this range of operating conditions that are important for many processes yet extremely hard to access experimentally.

Therefore, a method was established to measure hydromechanical stress that can be applied in large scale equipment at intense agitation and aeration. Details on the development of the measurement method are specified in Daub et al. [25]. The method is based on the theory of turbulent drop dispersion and uses the well established correlation of the maximum stable drop size d_max_ with maximum local energy dissipation rate ϵ_max_ for break-up controlled dispersions:

In an agitated tank, kinetic energy is introduced to the liquid by the action of the impeller. This energy dissipates in the reactor volume inhomogeneously. A maximum local value of energy dissipation, ϵ_max_, exists in the impeller region that defines the most severe action of the flow field on dispersed drops, bubbles or microorganisms. The ratio of maximum local energy dissipation rate to volume-averaged energy dissipation rate is given by:

(1) $$\phi =\frac{\epsilon_{\text{max}}}{\epsilon_{Ø}}$$

where ϵ_Ø_ is the average energy dissipation rate in the reactor volume per unit mass. ϵ_Ø_ is related to the volumetric power input via the density of the liquid phase ϵ_Ø_ = P/(ρV_L_). ϕ is constant for a given impeller, i.e. independent of agitation rate in single-phase operation [23]. It, therefore, characterizes a given reactor configuration in terms of the hydromechanical stress. ϵ_max_ can be related to the maximum stable drop size in a break-up controlled dispersion by [26]

(2) $$d_{\text{max}}=K_{1}\cdot {\left(\frac{\sigma }{\rho_{c}}\right)}^{3/5}\cdot \epsilon_{\text{max}}^{-2/5}\cdot {\left(1+K_{2}\cdot \frac{\eta_{d}}{\sigma }{\left(\epsilon_{\text{max}}\cdot d_{\text{max}}\right)}^{1/3}\right)}^{3/5}\cdot$$

with K_1_ = 0.23 [27] and K_2_ = 2.5 [26]. If d_max_ and ϵ_Ø_ are known, ϕ can be calculated iteratively from Eqs. 1 and 2. Since this equation models an effect of microturbulence, the constants should be independent of geometry and scale [26].

This theory is valid if the flow field is fully turbulent and the drop size is much smaller than the macroscale of turbulence Λ = 0.4 h (Λ/d_max_ > 10) and much larger than the microscale of turbulence *λ* = (*ν*^3^/*ϵ*)^1/4^ (d_max_/λ > 10). The flow field is fully turbulent when the Reynolds number is Re > 5∙10^3^ and Λ/λ > 150 [26].

There are different correlations available in literature that allow an estimation of ϕ for single-phase operation without aeration as a function of the impeller geometry for Rushton type impellers. These are listed in Table 1. Probably the most commonly used approach is McManamey’s equation [28] that estimates maximum energy dissipation by relating the total impeller power to the volume swept by the impeller. Jüsten et al. used this apprach to calculate the energy dissipation to circulation function EDCF [11-13]. Davies, 1985 [29] and [30] used the principle to correlate drop sizes for stirred tanks with different other mixing devices, [31] used this approach to correlate particle stress in stirred tanks. Kresta and Brodkey [32] recommend this approach “as the best practice estimate” to calculate maximum energy dissipation.

**Table 1:** Literature correlations

McManamey [28]	$\phi =\frac{4}{\pi }\cdot \frac{V_{L}}{d^{2}\cdot h}$	(3)
Okamoto et al. [33]	$\phi =0.85\cdot {\left(\frac{h}{D_{R}}\right)}^{-1.38}\cdot e^{-2.46\cdot d/D_{R}}$	(4)
Liepe et al. [26]	$\phi =\frac{0.1\cdot \pi^{3}}{\mathrm{Po}}\cdot \frac{V_{L}}{d^{2}\cdot h}$	(5)
Liepe et al. [26]	$\phi =0.11\cdot \pi^{3}\cdot \frac{V_{L}}{d^{3}}$	(6)

Literature correlations for the estimation of the ratio of maximum to volume-averaged energy dissipation rate ϕ for Rushton impellers for single-phase, unaerated operating conditions.

The experimental method based on the measurement of maximum stable drop sizes was successfully applied in a 3 m^3^ pilot scale reactor with 1.2 m inner diameter to investigate the influence of aeration on the maximum local energy dissipation rate. The results were reported in Daub et al. [24]. It was shown for impeller configurations B-1 and B-3 (for geometrical details see Table 2) that energy dissipation in the impeller region is much less intense for aerated operating conditions than for unaerated operating conditions when compared at equal volumetric power input. The ratio of maximum to volume-averaged energy dissipation rate was reduced by 64% for impeller setup B-1 and by 52% for impeller setup B-3, respectively. Thorough control experiments were presented concluding, that the interpretation of the data on the basis of the theory of break-up controlled drop dispersion is valid. Particularly the presence of coalescence as an explanation for increased drop sizes under aerated operating conditions was ruled out by experiments with and without aeration where dispersed phase concentration was varied up to factor 20 between lowest and highest concentration.

**Table 2:** Geometrical details of the reactors used, geometrical details of impeller configurations and ranges of turbulence parameters

**Geometrical details of reactors**								**Geometrical details of impellers**							**Turbulence parameters**					
**Reactor configuration**	**Nominal reactor size**	**D**_**R**_	**V**_**L**_	**H**	**Number of impellers**	**C**	**ΔC**	**d**	**h**	**w**	**d/D**_**R**_	**h/d**	**w/d**	**Po**	**n**	**Re**	**λ**	**λ/Λ**	**d**_**max**_**/λ**	**Λ/d**_**max**_
		**m**						**m**	**m**	**m**	**-**	**-**	**-**	**-**	**1/s**	**-**	**μm**	**-**	**-**	**-**
A-1	50 L	0.293	0.035	0.52	3	0.095	0.19	0.104	0.023	0.029	0.35	0.22	0.28	5.5	4.0 – 11.7	4.3 – 12∙10^4^	16 – 32	286 – 571	14 – 28	11 – 39
A-2								0.15	0.05	0.05	0.51	0.33	0.33	8	3.6 – 8.0	8.1 – 18∙10^4^	13 – 23	400 – 688	17 – 21	19 – 40
A-3								0.17	0.05	0.05	0.58	0.29	0.29	7.1	3.3 – 7.2	9.6 – 21∙10^4^	14 – 23	402 – 674	17 – 21	19 – 39
A-4								0.19	0.05	0.05	0.65	0.26	0.26	6.4	2.8 – 6.1	1.0 – 2.2∙10^5^	13 – 23	396 – 695	17 – 21	19 – 42
B-1	3 m^3^	1.2	2.4^a^	2.2	3	0.51	0.69	0.41	0.09	0.12	0.34	0.22	0.28	4.9	2.1 - 5.8	2.5 – 9.7∙10^5^	11 – 23	1.5 – 3.2∙10^3^	14 – 23	71 – 211
B-2								0.45	0.09	0.12	0.38	0.2	0.26	5	1.9 – 5.1	3.7 – 10∙10^5^	11 – 23	1.5 – 3.2∙10^3^	15 – 26	64 – 185
B-3								0.51	0.12	0.18	0.43	0.24	0.34	5.9	1.3 – 4.2	2.6 – 11∙10^5^	11 – 25	1.9 – 4.4∙10^3^	15 – 24	82 – 261
C-1	40 m^3^	2.8	30	5	3	0.92	1.8	1.19	0.22	0.29	0.42	0.19	0.24	4.7	0.8 – 2.3	1.1 – 3.3∙10^6^	11 – 22	3.9 – 8.3∙10^3^	13 – 24	163 – 652

All impellers were 6-bladed Rushton type impellers installed in a three impeller configuration. Power numbers were estimated with Eq. 10, except for setup B-1 and B-3 for which Power numbers could be measured.

^a^for 0.1 vvm aeration rate: 2.6 m^3^, see Daub et al. [24].

The same method will be applied in the current report with 8 different reactor configurations with Rushton type impellers in reactors of scales 50 L, 3 m^3^ and 40 m^3^ in a wide range of operating conditions (geometrical details are given in Table 2). It is the goal of this study to provide a broad data basis to demonstrate the applicability of this method to real life equipment of different scales, to characterize different reactor configurations with respect to hydromechanical stress as a basis for successful scale-up or scale-down of this parameter under aerated operating conditions and to test whether established literature correlations for the estimation of hydromechanical stress for different impellers can be applied with acceptable accuracy.

## Results and discussion

### Turbulence parameters

Parameter values for the turbulence parameters for the range of operating conditions used in the present study are presented in Table 2. The microscale of turbulence was calculated with maximum local energy dissipation rates based on Eq. 1 with values for ϕ also given in Table 2. All parameters are within the validity ranges for the application of the theory of turbulent drop dispersion (Re > 5∙10^3^_,_ Λ/d_max_ > 10, d_max_/λ > 10 and Λ/λ > 150).

### Drop size distributions in different scales

The shape of a drop size distribution reveals important insight into the nature of the processes that formed the distribution. It was argued that a similarity of drop size distributions for different operating conditions or different equipment is a strong indication that the microprocesses involved in forming the drop size distribution are comparable. Brown and Pitt [34], Chen and Middleman [35], Konno et al. [36], and Peter et al. [9], e.g., showed that a plot of a normalized drop diameter versus the cumulative volume distribution reveals invariant drop size distributions with respect to agitation rate and dispersion time. Normalization was done either by the Sauter mean diameter d_32_ or the maximum stable drop size d_max_. Invariance of normalized drop size distributions is referred to as self-similarity. Pacek et al. [37] point out that the observation of self-similarity might partly be due to a smoothing of fine differences by the cumulative distribution that is typically used for these plots. They argue that differences in drop size distributions might be clearer recognized in volume density distributions. Therefore, these will be used in this work.

**Figure 1:**
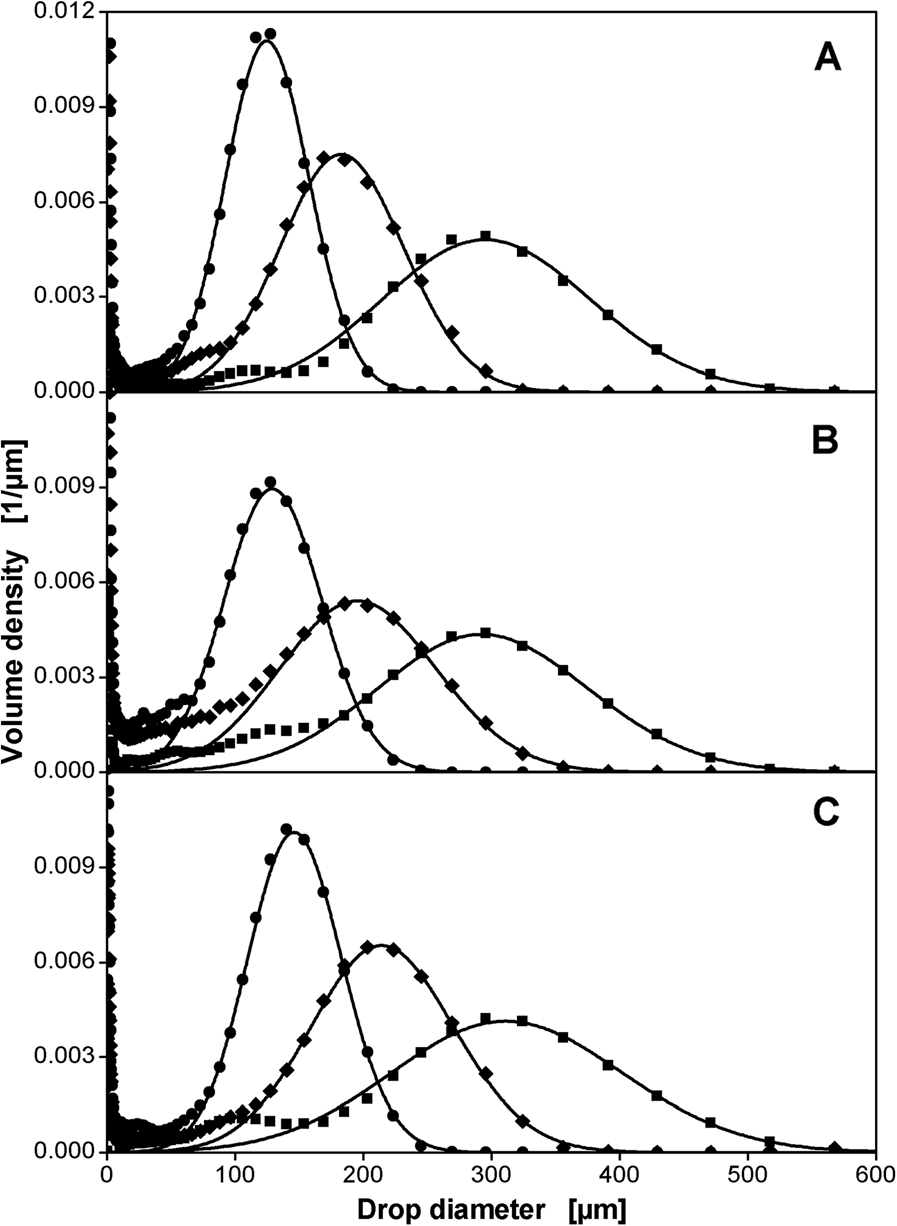
**Measured drop size distributions with similar maximum stable drop sizes in three different scales with different reactor configurations.** Dispersion of paraffin oil in 1 mM PO_4_-buffer at pH 7.3. **A:** A-2 (50 L reactor), **B:** B-3 (3 m^3^ reactor), **C:** C-1 (40 m^3^ reactor). See Table 2 for details on geometry. Aeration rates in all measurements 0.7 vvm. Symbols: measured values for agitation rates: A: (●) 8.0 1/s (11 kW/m^3^), (♦) 5.2 1/s (4.4 kW/m^3^), (■) 3.6 1/s (1.3 kW/m^3^); B: (●) 3.7 1/s (6.2 kW/m^3^), (♦) 2.3 1/s (1.7 kW/m^3^), (■) 1.3 1/s (0.6 kW/m^3^); C: (●) 1.8 1/s (2.0 kW/m^3^), (♦) 1.3 1/s (0.9 kW/m^3^), (■) 0.8 1/s (0.2 kW/m^3^). Solid lines: fitted normal distributions.

Volume density distributions for all three reactor sizes used in this study are compared with each other in Figure 1. Configuration A-2 is shown for the 50 L reactor, B-3 for the 3 m^3^ reactor and C-1 for the 40 m^3^ reactor. It must be emphasized that the reactor configurations are not geometrically similar. The drop size distributions shown were chosen to represent comparable maximum stable drop sizes in all three scales. Agitation rates and volumetric power inputs differed strongly for the experiments shown (values are given in the caption of Figure 1). The three drop size distributions with the smallest drop sizes, e.g., did occur at 11 kW/m^3^ in the 50 L reactor (A-2), at 6.2 kW/m^3^ in the 3 m^3^ reactor (B-3) and at 2.0 kW/m^3^ in the 40 m^3^ reactor (C-1). The drop size distributions are governed by a strong main peak that can be fit very well by a normal distribution (solid lines in the graph). There is a slightly increased tendency to bimodal distributions with increasing scale. This results in a reduced maximum value of the main peak of the volume density distributions because the integral of the volume density distribution is unity by definition. A second peak in the small diameter range, therefore, reduces the area of the main peak. Very small drops in the range < 50 μm are also present that might be daughter drops that developed during break up of larger drops and did not coalesce to larger droplets any more. These small droplets are not relevant for the subject of this work as described in [9] and [25]. Table 3 compares characteristic values for the main peaks of the distributions that were calculated from the fitted normal distributions. The ratio of d_32_/d_max_ falls within a narrow range of 0.56 to 0.61 for all the distributions from the three scales. This shows that the drop size distributions for the different operating conditions and scales are self-similar. The values found for d_32_/d_max_ are well in agreement with data from other groups found in break-up controlled single-phase experiments without aeration. Calabrese et al. [38], e.g., found values of 0.6 for moderately viscous dispersed phases.

**Table 3:** **Comparison of maximum stable drop sizes d**_**max **_**and Sauter mean diameters d**_**32 **_**for all three scales**

**Reactor configuration**	**Agitation rate**	**Maximum stable drop size d**_**max**_	**Sauter mean diameter d**_**32**_	**d**_**32**_**/d**_**max**_
	**1/s**	**μm**	**μm**	**-**
A-2	8.0	207	125	0.60
	5.2	305	183	0.60
	3.6	500	296	0.59
B-3	3.7	226	129	0.57
	2.3	350	196	0.56
	1.3	501	293	0.59
C-1	1.8	238	146	0.61
	1.3	355	215	0.61
	0.8	539	312	0.58

Operating conditions were chosen to result in similar maximum stable drop sizes for the different scales.

Maximum stable drop sizes calculated from these distributions are indicators for hydromechanical stress. To perform a scale-up or scale-down of hydromechanical stress it is necessary to correlate the maximum stable drop size with operating conditions. This is the focus of the following paragraphs.

### Correlation of maximum stable drop size with impeller tip speed u_t_

**Figure 2:**
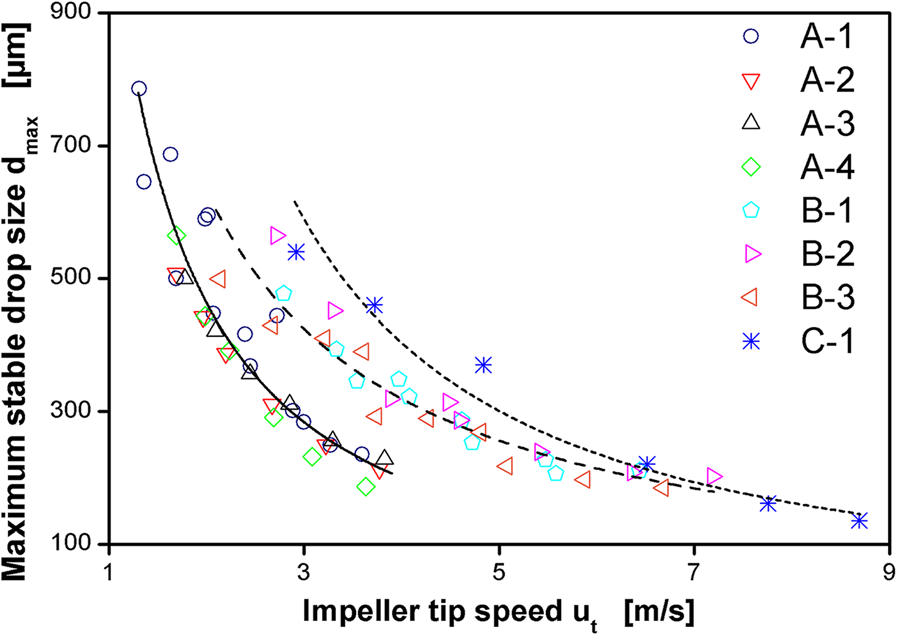
**Maximum stable drop size as a function of impeller tip speed u**_**t **_**for all impellers as indicated in the legend (impeller geometries according to Table **2**).** Lines indicate power law fit for different scales: solid: 50 L scale, dashed: 3 m^3^ scale, small dashed: 40 m^3^ scale. Dispersion of paraffin oil in 1 mM PO_4_-buffer at pH 7.3. Maximum stable drop size measured after 3 h agitation. Aeration rates in all measurements 0.7 vvm.

Maximum stable drop sizes for the different scales and different impellers are compared in Figure 2 at an aeration rate of 0.7 vvm (volume gas/volume liquid/minute). The data for the different impellers within the 50 L and the 3 m^3^ scale are in good agreement with each other. This shows that a correlation of hydromechanical stress with impeller tip speed u_t_ = π∙n∙d gives reasonable results as long as the scale is not changed. However, a comparison of the results for the different scales shows that a scale-up with constant impeller tip speed will not result in comparable values of d_max_. Therefore, the levels of hydromechanical stress in the different scales will be different if u_t_ is kept constant. This is in accordance with the results of Jüsten et al. [13] who showed that the fragmentation of *Penicillium chrysogenum* mycelium can be correlated well with impeller tip speed for different impeller geometries within one scale but not for different scales.

**Figure 3:**
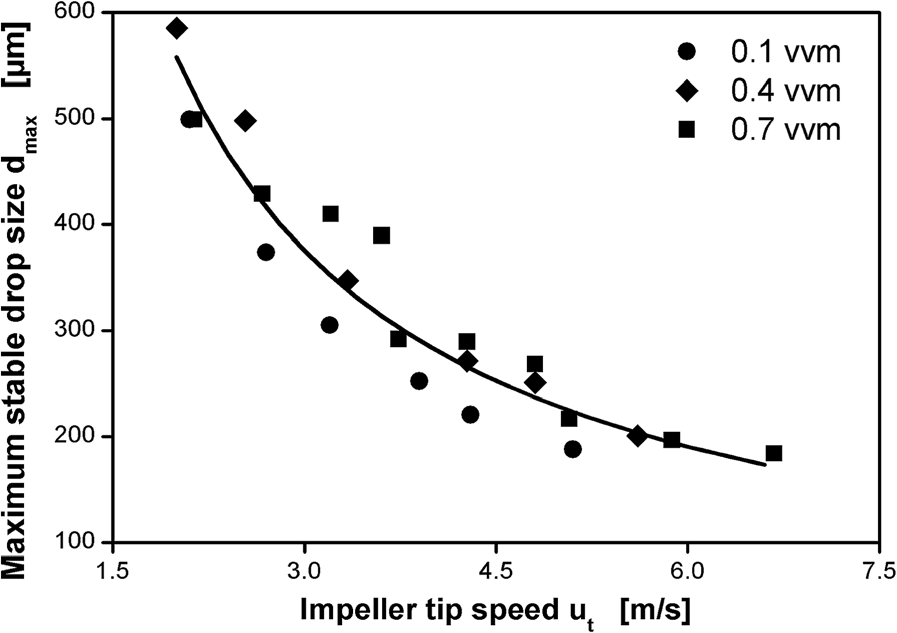
**Correlation of maximum stable drop size with impeller tip speed for reactor configuration B-3 (3 m**^**3 **^**scale) for different aeration rates.** Dispersion of paraffin oil in 1 mM PO_4_-buffer at pH 7.3. Maximum stable drop size measured after 3 h agitation. Solid line indicates fitted power law curve.

Figure 3 shows the maximum stable drop sizes versus impeller tip speed for reactor configuration B-3 operated with different aeration rates from 0.1 vvm to 0.7 vvm together with a power function correlation of the data. Most data for the highest aeration rate of 0.7 vvm lies at or above the fitted line while most data for the lowest aeration rate of 0.1 vvm lies below the fitted line. Generally, at the same impeller tip speed, higher aeration rates yield larger maximum stable drop sizes. This shows that there is an influence of aeration on maximum stable drop size that cannot be incorporated using u_t_.

Especially in industrial practice impeller tip speed is frequently applied as a correlator for hydromechanical stress. Margaritis and Zajic [39] estimate that 20% of the fermentation processes in industry are scaled up based on this rule. The results shown here, in accordance with earlier analyses on the value of impeller tip speed for scale-up of processes [13,15,40,41], clearly show that impeller tip speed is not well suited to correlate hydromechanical stress in fully turbulent aerated stirred tanks.

### Correlation of maximum stable drop size with volumetric power input

**Figure 4:**
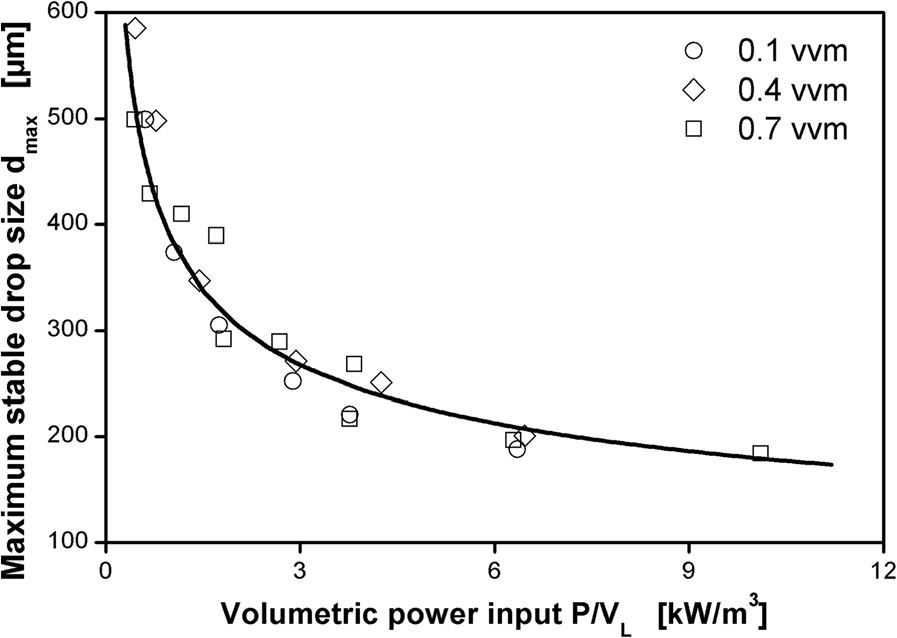
**Correlation of maximum stable drop size with volumetric power input for reactor configuration B-3 (3 m**^**3 **^**scale) for different aeration rates.** Dispersion of paraffin oil in 1 mM PO_4_-buffer at pH 7.3. Maximum stable drop size measured after 3 h agitation. Solid line represents theoretical prediction based on Eqs. 1 and 2 with ϕ = 6.9.

The theory on drop break-up (as described in the background section) suggests a correlation of maximum stable drop size for a given impeller with power per unit mass ϵ_Ø_ or equivalently volumetric power input P/V_L_. It was already reported in Daub et al. [24] for configurations B-1 and B-3 that if results are compared on the basis of aerated volumetric power input the aeration rate has no relevant influence on the maximum stable drop size in the investigated range. Hence, with aerated volumetric power input as the correlating parameter, the influence of aeration on energy dissipation is directly reflected. This means turbulence intensity scales directly with volumetric power input also in the aerated case. This is demonstrated in Figure 4 with the same data as in Figure 3. The solid line shows that the data is well in accordance with the theoretical prediction from Eqs. 1 and 2 (ϕ = 6.9).

For cell culture processes, not only the effect of maximum energy dissipation induced by the flow field is relevant for cell damage [42,43]. More importantly, bubble formation at the sparger and bubble rupture at the liquid surface are known to be the major cause for cell death by hydrodynamic forces in these processes [44-46]. Attachment of cells to bubbles plays an important role in the lethal effects of bursting bubbles in cell culture processes [47,48]. There is no evidence that these effects may have an influence on the maximum stable drop size. Measurements were conducted with aeration rates varied from 0.1 vvm to 1 vvm. If bubble rupture had a considerable influence on the maximum stable drop size a correlation of maximum stable drop size with aeration intensity migh be expected. Additionally, when unaerated and aerated data were compared in a previous publication [24], maximum stable drop size was smaller without aeration than with aeration (with the same volumetric power input). Hence, it can be concluded that the experimental results shown in this study are not influenced by the effects of bursting bubbles.

**Figure 5:**
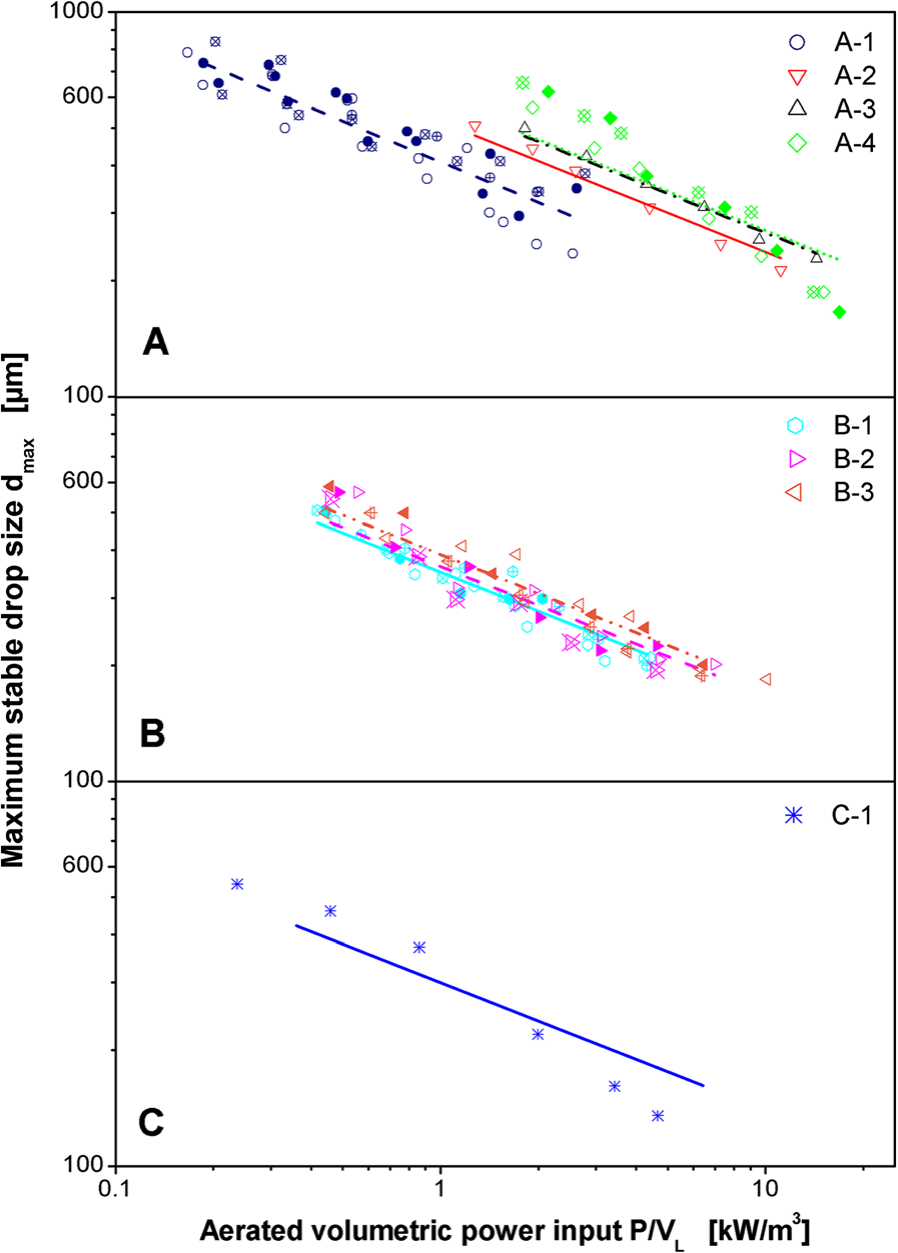
**Maximum stable drop sizes measured in three different scales: A: 50 L, B: 3 m**^**3**^**, C: 40 m**^**3**^**.** Dispersion of paraffin oil in 1 mM PO_4_-buffer at pH 7.3. Maximum stable drop size measured after 3 h agitation. Symbols for different reactor configurations as indicated in legend of Figure 2 (geometries according to Table 2). Code for different aeration rates: +-center: 0.1 vvm, full symbol: 0.4 vvm, open symbol: 0.7 vvm, x-center: 1.0 vvm.

Figure 5 shows the correlation of maximum stable drop sizes for all three reactor scales with volumetric power input. The results for configurations A-1 and A-4 in the 50 L reactor and for B-2 in the 3 m^3^ reactor again demonstrate that the influence of aeration for each impeller type is well reflected by correlating the data with aerated volumetric power input. Therefore, the data for the reactor configurations where aeration rate was not varied can be regarded as representative for these reactor configurations. The data for all scales and all impeller geometries follow generally the prediction of the theory for turbulent drop break-up. This is indicated by the lines in Figure 5. These were calculated on the basis of Eqs. 1 and 2 by fitting the value of ϕ to the whole data set of each impeller by means of the least squares method.

The measurements clearly discriminate between the different impeller configurations in the 50 L reactor. The larger the impeller, the larger the maximum stable drop size at a given volumetric power input. This is equivalent with a decrease in the ratio of maximum to specific energy dissipation rate with increasing impeller size and consistent with existing literature data for unaerated operating conditions [28].

The characteristics of the impellers used in the 3 m^3^ reactor are relatively similar in relation to the measurement accuracy for the maximum stable drop size and the impellers can hardly be distinguished. Nevertheless, a sequence of the three impeller configurations is apparent that is in accordance with the results for the 50 L reactor. The smallest impeller B-1 produces the smallest maximum stable drop sizes at a given volumetric power input. The larger impeller B-2 with the same impeller blades as B-1 produces larger drops and the largest impeller with larger impeller blades B-3 results in the largest maximum stable drop sizes at a given volumetric power input.

For the 40 m^3^ reactor, the data suggests a higher slope than predicted by the classic theory of drop dispersion that is represented by Eqs. 1 and 2. However, the data is relatively scarce because the reactor was only available for a short period of time. The quality of the fit of the data by the correlation is compared for all reactor configurations in Table 4. The quality of the fit is measured by the standard deviation of the relative difference between measured value and calculated value for d_max_. For all reactor configurations, including the 40 m^3^ reactor, the values are below the standard deviation of d_max_ for independent experiments which was determined to approx. 10% [25]. That means the deviation between measurement and model has a similar magnitude as the deviation between independent experiments. It is, therefore, not possible to clearly distinguish between a systematic deviation of the results from the classic theory of drop dispersion and measurement inaccuracy for a relatively small set of data as for the 40 m^3^ reactor.

**Table 4:** Quality of the fit for all 8 reactor configurations

**Reactor configuration**	**A-1**	**A-2**	**A-3**	**A-4**	**B-1**	**B-2**	**B-3**	**C-1**
Standard deviation [%]	6.1	2.0	1.7	9.0	3.9	5.1	4.0	6.7

Standard deviations of the relative difference between measured values for d_max_ and calculated values based on Eqs. 1 and 2 (values for ϕ see Table 5) were used as a measure for the quality of the fit.

Turbulence intermittency might explain an increased slope for d_max_. This extension of the classic theory of drop dispersion takes into account the intermittent character of fine-scale turbulence as laid out by Baldyga and Podgorska [27] and Baldyga et al. [49]. In this concept, ϵ_max_ is not taken as a constant but as a stochastic variable that fluctuates about its mean value. The theory predicts that rare but strong bursts of high energy become more and more important for the evolution of the maximum stable drop size with increasing dispersion time. This results in a long-term drift of maximum stable drop sizes towards smaller drops. As a consequence, the exponent on maximum energy dissipation rate becomes time-dependent with values up to -0.62 for very long dispersion times [49]. Additionally, intermittency is stronger for higher Reynolds numbers [50] and, therefore, the effect becomes increasingly important with increasing scale.

All data presented in this study were measured between 100 and 180 min of dispersion time. The maximum stable drop sizes measured were essentially constant during this time span in all reactors. Examples for this were shown in Daub et al. [25]. Extending the experimental time to up to 9 h yielded a further decrease of the maximum stable drop size in the range of 10% compared to the value at 3 h dispersion time in the 3 m^3^ reactor. This is qualitatively in agreement with the prediction of the effect of intermittent turbulence, but the extend of the effect is relatively small and comparable to the reproducibility of the measured values in independent experiments. The data for the 3 m^3^ reactor follows equally well the classic theory of drop dispersion as the data for the 50 L reactor. It must be assumed that the scale-effect on the slope cannot be resolved with the applied measurement method because it is below the reproducibilty of the experimental method. If the measurement accuracy in the 40 m^3^ reactor is similar to that in the other scales and taking into account that the effect of turbulence intermittency on the slope was not strong enough to be detected for the other reactor configurations it seems justified to assume that the differences between measurement and model for the 40 m^3^ reactor rather reflect measurement inaccuracy than the effect of intermittency on the slope. We, therefore, simplify the analysis and restrict the value of the slope to that of the classic theory of drop dispersion.

The data for the largest impeller in the 50 L reactor (A-4) exhibits a higher slope than expected for all aeration rates. This impeller has an extreme geometry with very large d/D_R_-ratio of 0.65 and large impeller blades. The distance between the impeller tip and the baffles is only 0.02 m. This probably gives rise to a nonstandard flow-field which may result in a modification of the turbulence characteristics. Additionally, the calculated values for power input based on Eq. 8 might possess a larger error than for the other impellers that are closer to standard geometry. The impellers for A-2, A-3 and A-4 have the same impeller blades but different impeller diameters. A-3 generates larger maximum stable drop sizes at the same power per unit volume than A-2. If this trend is extrapolated to A-4 than larger maximum stable drop sizes may be expected for A-4 than for A-3 at the same volumetric power input. The values for the lower range of power inputs for A-4 up to 4 kW/m^3^ are in agreement with this expectation but the data for the higher power inputs tend towards smaller maximum stable drop sizes than expected. By interpreting the data on the basis of Eqs. 1 and 2 the value of ϕ for this impeller might be overestimated in comparison to the other impellers.

### Estimation of maximum local energy dissipation rate ϵ_max_ and the ratio of maximum to specific energy dissipation rate ϕ

**Figure 6:**
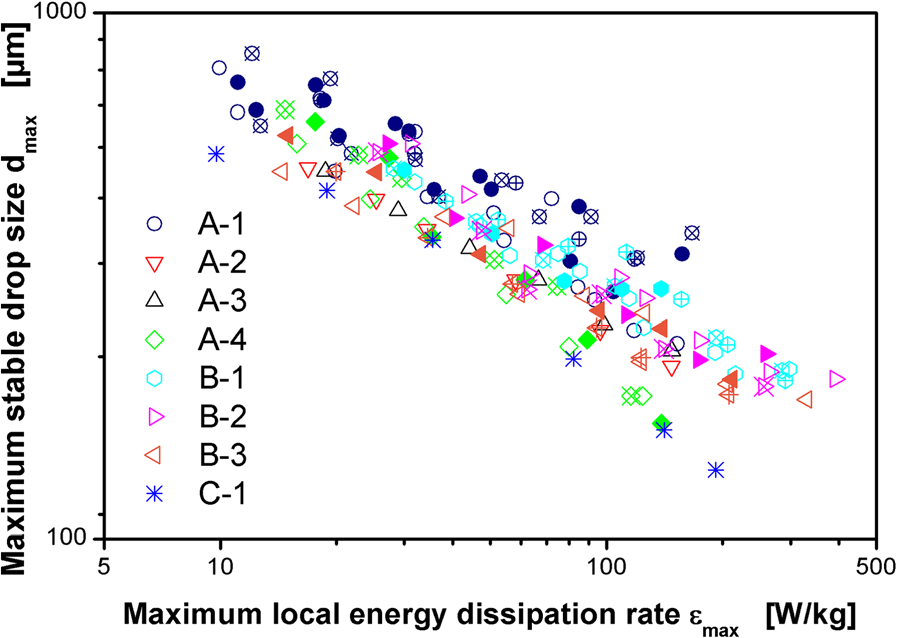
**Maximum stable drop size as a function of maximum local energy dissipation rate ϵ**_**max**_**.** ϵ_max_ calculated from ϵ_Ø_ with ϕ = ϵ_max_/ϵ_Ø_ based on the equation of McManamey [28] for non-aerated conditions. Dispersion of paraffin oil in 1 mM PO_4_-buffer at pH 7.3. Maximum stable drop size measured after 3 h agitation. Geometries for different reactor configurations according to Table 2. Code for different aeration rates: +-center: 0.1 vvm, full symbol: 0.4 vvm, open symbol: 0.7 vvm, x-center: 1.0 vvm.

**Figure 7:**
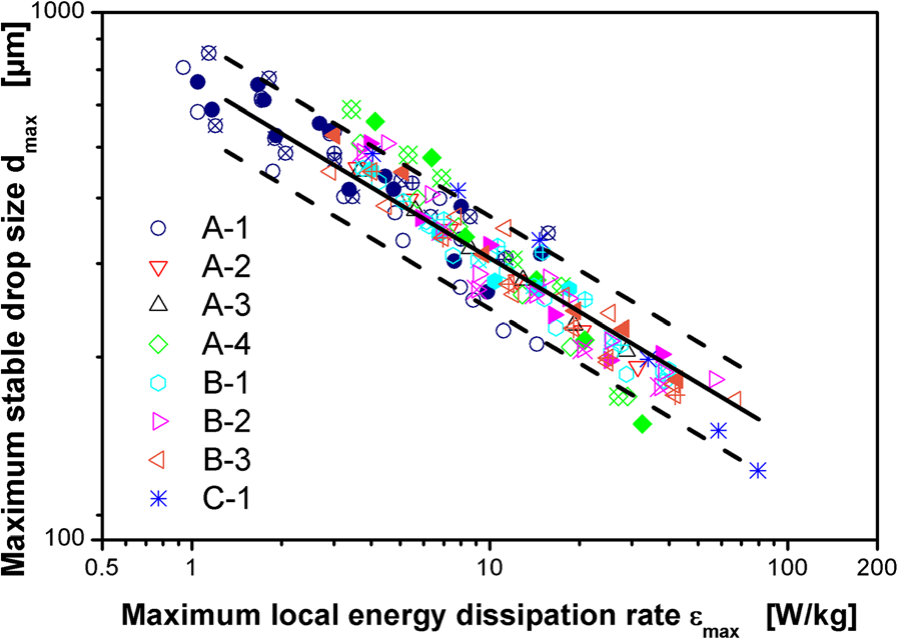
**Maximum stable drop size as a function of maximum local energy dissipation rate ϵ**_**max**_**.** ϵ_max_ calculated on the basis of ϕ = ϵ_max_/ϵ_Ø_ based on the measurements. Dispersion of paraffin oil in 1 mM PO_4_-buffer at pH 7.3. Maximum stable drop size measured after 3 h agitation. Geometries for different reactor configurations according to Table 2. Code for different aeration rates: +-center: 0.1 vvm, full symbol: 0.4 vvm, open symbol: 0.7 vvm, x-center: 1.0 vvm. Solid line: Theoretical prediction based on Eqs. 1 and 2, values for ϕ from Table 5. Dashed lines: plus/minus 20% deviation from theoretical prediction.

The literature correlations for the ratio of maximum to specific energy dissipation rate ϕ given in Table 1 can be used to calculate the maximum local energy dissipation rate ϵ_max_ for different operating conditions and reactor geometries. It must be emphasized that these correlations were derived for single-phase, unaerated operating conditions and not for aerated operating conditions. However, up to now the only practical way to estimate ϕ for different reactor configurations for aerated operating conditions was to assume that these correlations can be applied also in the presence of aeration. This was first supported by the data of Bourne [51] that is based on a chemical method to measure micromixing efficiency. Bourne [51] came to the conclusion that ϕ is not influenced by aeration. Fort et al. [52] report on a roughly 20% reduction of turbulence intensity in the presence of aeration. However, their data is based on the measurement of pressure fluctuations at constant agitation rate. There is no clear conclusion with regard to the influence of aeration on ϕ. The data presented in Daub et al. [24] clearly shows that ϕ is reduced by aeration based on the same measurement technique that is used in this study. The relation of these results to the findings of Bourne [51] and newer literature data incorporating similar methods are discussed in Daub et al. [24]. As an example for the value of the correlations from Table 1 for aerated operating conditions, Figure 6 compares all data obtained in this work with ϵ_max_ calculated with the correlation of McManamey [28]. This equation was used because it might be the most common correlation to estimate ϵ_max_[24]. Given the simplicity of this type of correlation, the broad spectrum of impeller geometries used in the experiments and the broad range of scales applied, the results are in reasonable agreement. The prediction from this simple correlation is probably accurate enough for crude estimations in industrial practice.Figure 7 shows the data for all impeller configurations and all operating conditions for the three scales with the maximum local energy dissipation rate calculated with the values for ϕ based on the experimental data and Eqs. 1 and 2. Most of the data lies within ±20% around the prediction as indicated by the solid and dashed lines in Figure 7. A very accurate correlation of the data is achieved. This emphasizes the importance of the experimental method applied for scale-up and scale-down studies of hydromechanical stress in aerated stirred tanks.

Table 5 allows an analysis of the main factors that influence the value of ϕ. The table shows the values for ϕ that were derived from the aerated experiments and the values calculated with the different literature correlations from Table 1. The absolute values for ϕ for each impeller differ strongly for the different correlations and in comparison to the values that are based on the measurements. This was expected and already discussed in Daub et al. [24]. Despite the differences in the absolute values, the relative order of the impeller configurations within the 50 L and the 3 m^3^ scales is the same for the measurements as for all the different correlations. That means the influence of geometry within one scale is qualitatively well predicted by the correlations.

**Table 5:** Comparison of experimental values for ϕ and values from literature correlations

**Reactor configuration**	**Results from experiments with aeration**	**Correlations for single-phase, unaerated operation**			
		**McManamey (1979)**	**Okamoto (1981)**	**Liepe (1988)**	**Liepe (1988)**
		**Eq. (**3**)**	**Eq. (**4**)**	**Eq. (**5**)**	**Eq. (**6**)**
	**ϕ [−]**	**ϕ [−]**	**ϕ [−]**	**ϕ [−]**	**ϕ [−]**
A-1	5.7	60	12	26	35
A-2	2.8	13	2.8	4.0	12
A-3	2.0	10	2.4	3.5	8.1
A-4	1.9	8.2	2.0	3.1	5.8
B-1	9.9	67	13	33	40
B-2	8.1	56	12	27	30
B-3	6.9	33	7.2	17	21
C-1	17	41	10	21	20

Values for ϕ calculated for all 8 reactor configurations from the experimental data for maximum stable drop size from aerated experiments in comparison with results from literature correlations (Table 1) for single-phase, unaerated operation.

The absolute values for ϕ that were derived from the measurements are small compared to the values from literature correlations. It was shown in Daub et al. [24] that a comparison of maximum stable drop sizes under aerated and unaerated operating conditions for reactor configurations B-1 and B-3 reveals a strong attenuation of turbulence intensity by the presence of air. ϕ was reduced by aeration by 64% for B-1 and by 52% for B-3 when compared with unaerated operating conditions on the basis of equal volumetric power input. The low values for ϕ found for the data presented in this study fit well into this pattern and support this finding.

If different scales are compared with each other the measured values for ϕ suggest a scale-effect with higher values for ϕ in larger scales. A-1 and B-1 for example are close to geometric similarity and the literature correlations predict similar values for ϕ for the two impeller configurations. McManamey’s [28] correlation, e.g., predicts that the ratio of the values of ϕ for B-1 compared to A-1 should be 1.1. However, the ratio of the values of ϕ derived from the measurements is 1.7. That means that hydromechanical stress at equal volumetric power input is higher in the 3 m^3^ reactor than in the 50 L reactor although the impeller geometries are close to geometric similarity. A comparison of the data for the 40 m^3^ reactor with the 3 m^3^ reactor also shows this scale-effect. C-1 has a larger diameter impeller than B-1 with similar sized impeller blades in relation to the reactor diameter. Within one scale this combination results in a lower value for ϕ for the larger impeller (e.g. A-2 in comparison with A-1). This is also predicted by the correlation of McManamey [28] that predicts a ratio of the values of ϕ for C-1 compared to B-1 of 0.6. The experimental data however gives a ratio of 1.7 for C-1 compared to B-1, i.e. hydromechanical stress is higher at the same volumetric power input in C-1 than in B-1. The classic theory of drop dispersion as expressed in Eqs. 1 and 2 does not predict a scale-dependence of maximum stable drop size for geometrically similar reactor configurations for aerated operating conditions. However, Baldyga et al. [49] show for inviscid drops in unaerated dispersions that turbulence intermittency can explain a scale-dependence of d_max_ that leads to smaller drops in larger scales. The extent of the scale-dependence is related to dispersion time through the “multifractal scaling exponent”. A parameter that is not readily available for practical applications. For long dispersion times the theory predicts a dependence in the form d_max_ ~ D_R_^-0.543^[49]. If this is the case and the data is still interpreted on the basis of the classic theory of drop dispersion this will result in an apparently higher value of ϕ for large reactors. If the case of inviscid drops is considered with d_max_ ~ ϵ_max_^-0.4^ then ϕ ~ D_R_^0.543^ would result following the theory of Baldyga. The proportionality ϕ ~ D_R_^0.543^ can be used to calculate the theoretical ratios of ϕ for the different reactor scales used in this study assuming geometric similarity: the ratio of ϕ for the 3 m^3^ reactor compared to the 50 L reactor is 2.2. The calculated ratio of ϕ for the 40 m^3^ reactor compared to the 50 L reactor is 3.4 and the ratio of ϕ for the 40 m^3^ reactor compared to the 3 m^3^ reactor is 1.6. These differences are in reasonable agreement with the differences seen in the experimental results for ϕ. Although the effect of intermittency on the exponent on energy dissipation rate could not be resolved with the measurement method applied in the experiments, the effect of intermittency on ϕ is strong and must be incorporated in the analysis.

For practical applications it is desirable to estimate ϕ based on a simple engineering correlation instead of conducting time consuming and costly experiments (particularly in large scale). It is possible to get a first approximation by applying one of the correlations from Table 1. However, these only model the effect of geometry on ϕ for unaerated operating conditions. It would be favourible to generalize these correlations by additionally incorporating the effects of aeration and scale. It is clear that a correlation based on the limited set of data presented in this study can only be preliminary and approximate. Nevertheless, it might be helpful for practitioners and will hopefully inspire further work to elaborate the results presented in this work. The impeller geometry can be incorporated using, e.g. the equation of McManamey [28] for single-phase, unaerated operating conditions (Eq. 3, Table 1). The results presented in Daub et al. [24] indicated already that the effect of aeration on ϕ is geometry-dependent, i.e. turbulence attenuation by aeration is stronger for impellers that exhibit larger values of ϕ under unaerated conditions. The data in this study strongly support the presence of this phenomenon. The results can only be correlated satisfactorily when the geometry-dependence of the effect of aeration on ϕ is considered. This can be done in the form ϕ ~ (ϕ_unaerated_)^a^ where “a” is a constant. The effect of scale can be estimated by ϕ ~ D^0.543^ based on the work of Baldyga et al. [49]. This results in the following correlation:

(7) $$\phi =2.3\cdot \phi_{\mathrm{unaerated}}^{0.34}\cdot D_{R}^{0.543}$$

**Figure 8:**
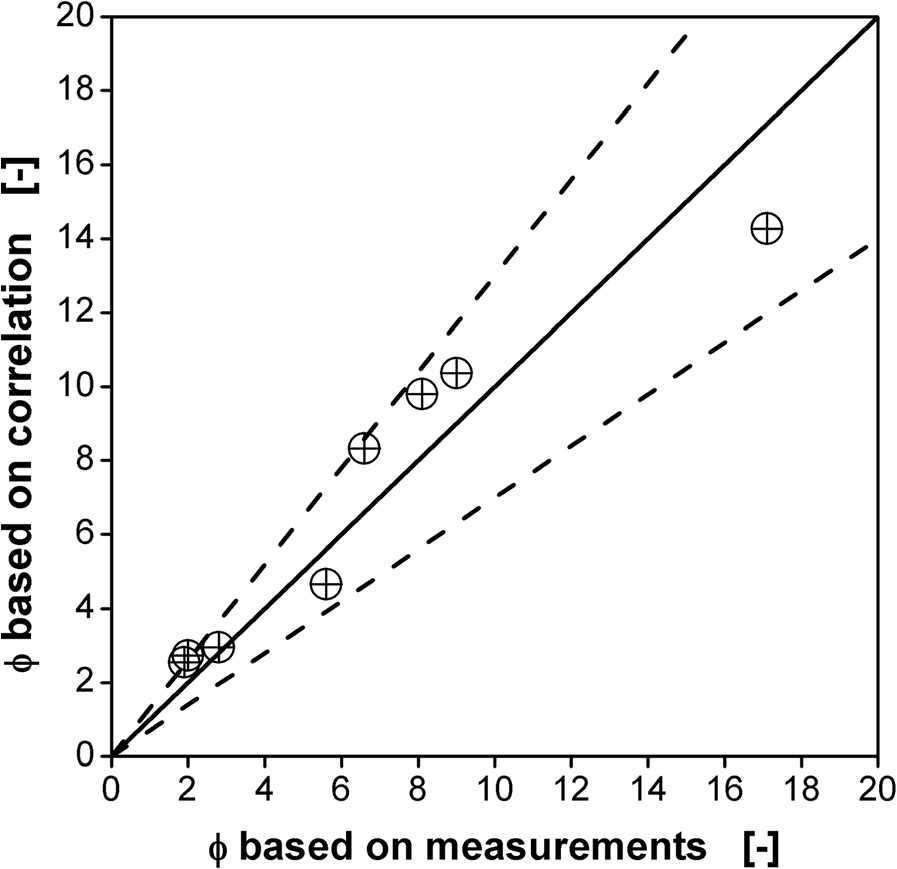
**Parity plot of measured and calculated values of ϕ for all impeller configurations and scales.** Measured values as given in Table 5, calculated values based on Eq. 7 with ϕ_unaerated_ calculated with Eq. 3. Dotted lines indicate +/- 30%-lines.

The proportionality constant and the exponent on ϕ_unaerated_ were found by means of least squares fitting to the values of ϕ based on the measurements of maximum stable drop size (Table 5). ϕ_unaerated_ was calculated based on the impeller swept volume (Eq. 3). Figure 8 shows the excellent agreement of the results calculated with Eq. 7 with the values based on the measured data for maximum stable drop size for all impeller geometries and scales from 50 L to 40 m^3^.

## Conclusions

For the first time, results from drop dispersion experiments in aerated stirred tanks were presented that cover a broad range of operating conditions, impeller geometries (Rushton impellers) and reactor scales of 50 L, 3 m^3^ and 40 m^3^ volume. A comparison of the volume density distributions in the three different scales show that the drop size distributions are self-similar and that d_32_/d_max_ for the aerated dispersions are in the same range as reported by other groups for single-phase, unaerated dispersions e.g. [38]. It was shown that the influence of aeration and scale on hydromechanical stress is not considered correctly when using impeller tip speed as the correlator. The influence of aeration for each impeller type is well reflected by correlating the data with aerated volumetric power input. This is in accordance with the classic theory of break-up controlled drop dispersion if the ratio of maximum to volume averaged energy dissipation rate ϕ is independent of the operating conditions. Absolute values for ϕ that were calculated for each impeller based on literature correlations for unaerated operating conditions differ strongly for the different correlations and in comparison to the values derived from the measurements with aerated operating conditions. The relative order of the impellers within each scale is the same for all correlations for unaerated operating conditions as for the values that are based on the drop size measurements for aerated operating conditions. Hence, the behavior of the impellers relative to each other within each scale is qualitatively well predicted by the correlations even though they are strictly valid only for unaerated operating conditions. The low values for ϕ found for the data presented in this study support the finding reported in Daub et al. [24] that hydromechanical stress is strongly reduced (ϕ is reduced by approx. 60%) for aerated operating conditions compared to unaerated operating conditions at the same volumetric power input. Comparison of data in the different scales shows that there is a scale effect that results in higher values for ϕ in larger reactors. This behavior is not covered by the classic theory of turbulent drop dispersion but is in good agreement with the theory of turbulence intermittency that predicts an up to 3.4 times larger value for ϕ in the 40 m^3^ reactor than in the 50 L reactor. The data for all impeller configurations and all aeration rates for the three scales correlate very well when calculated values for ϕ based on the measured values for d_max_ are used to calculate the maximum local energy dissipation rate. Most of the data lies within 20% around the theoretical prediction from the classic theory of drop dispersion when these values for ϕ are used. A correlation of the data for all scales and all impeller configurations in the form ϕ = 2.3∙(ϕ_unaerated_)^0.34^∙(D_R_)^0.543^ is suggested that successfully models the influence of impeller geometry, aeration and scale on ϕ for aerated operating conditions. Incorporating the effects of aeration and scale on hydromechanical stress is beneficial for a successful scale up or scale down of this parameter. This can be done by applying the suggested correlation or by measuring hydromechanical stress with the experimental method used in this study.

## Materials and methods

### Reactor and impeller configurations

**Figure 9:**
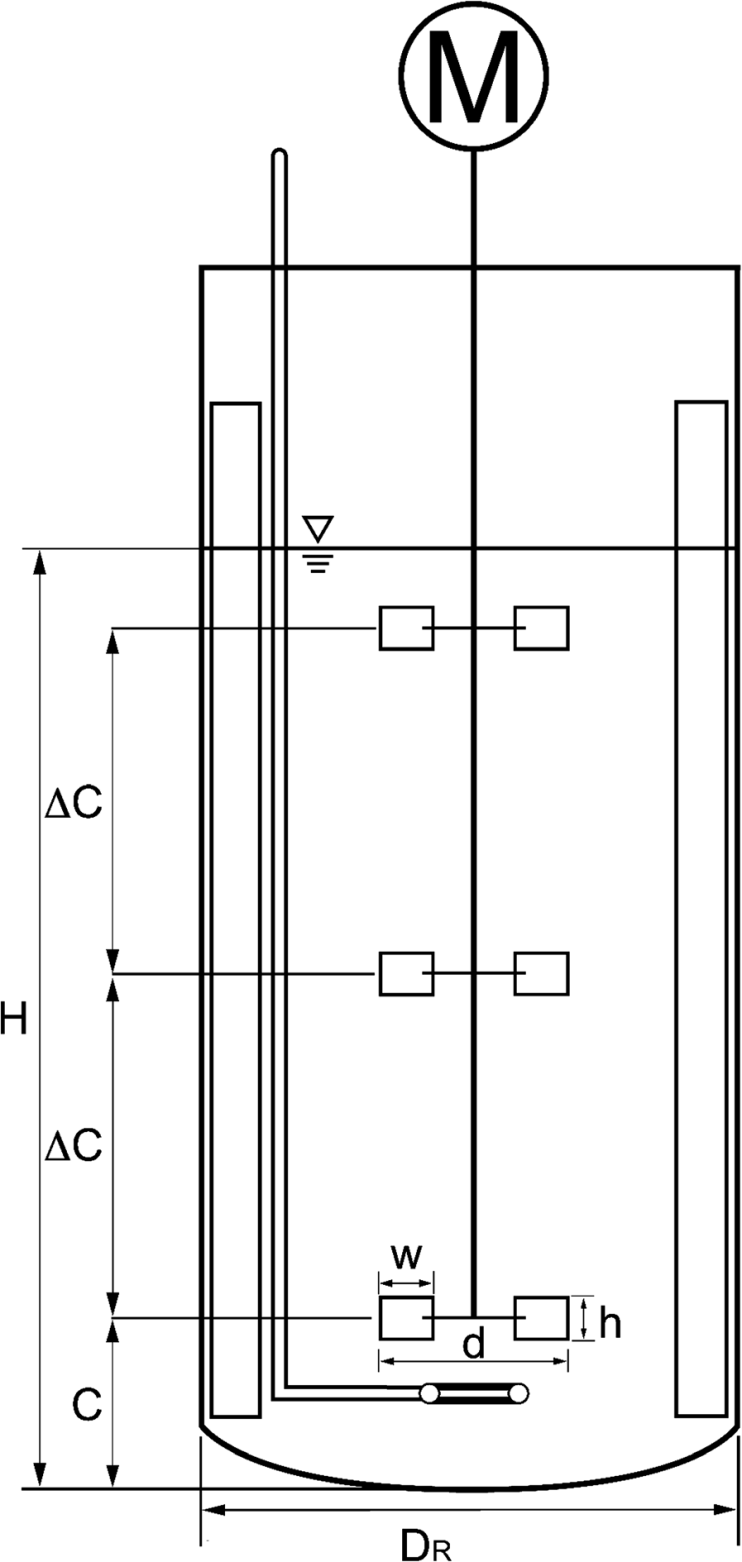
Schematic drawing of the reactors used for the experiments.

Experiments were conducted in stainless steel vessels. A schematic drawing of the reactors is depicted in Figure 9. Geometrical details of the tanks are given in Table 2. The 50 L and 3 m^3^ reactors were equipped with 4 baffles of width D_R_/10. The 40 m^3^ reactor had cooling pipes installed that act as baffles. Due to the size of the cooling pipes it can be assumed that the influence of the pipes on the flow field is comparable to conventional baffles [26,53]. The filling volume was chosen to result in equivalent ratios of unaerated liquid height to tank diameter of approx. 1.8 in all three scales. The sampling ports were at different positions in all three reactors. It was not possible to align the sampling ports in the three reactors because additional ports could not be installed. Since the dispersion is break-up controlled it can be assumed that the reactor is homogeneous with respect to the drop size distribution. The sampling positions should, therefore, not be relevant. This was tested in the 3 m^3^ tank where a second sampling point was available on the bottom of the reactor and direct sampling through the manway opening at the top was also possible. Comparison of samples from these alternative sampling positions with the results from the standard sampling point showed no influence of the sampling position on the measured drop size distribution (data not shown). In the 50 L reactor the sampling port was located at 0.24 m from the tank bottom between the middle and the upper impeller. In the 3 m^3^ reactor, the sampling port was at 2.1 m from the bottom close to the unaerated liquid surface above the upper impeller and in the 40 m^3^ reactor it was at 3.5 m from the tank bottom above the second impeller. All sampling ports were half way between two baffles or cooling pipe installations, respectively. Rushton type 6-bladed impellers with different geometries were used in the experiments. The geometrical details are given in Table 2. All impellers were installed in a three impeller configuration which is typical for high aspect ratio reactors used, e.g., in the fermentation industries.

### Measurement of drop size distributions and maximum stable drop size

The development of the experimental procedure to measure drop size distributions and maximum stable drop size including the rationale for the dispersed and continuous phases used for the dispersion experiments were reported in Daub et al. [25]. Details on the preparation of the 50 L reactor were also given there. Experimental details specific for the 3 m^3^ reactor were presented in Daub et al. [24]. The experiments in the 40 m^3^ reactor were conducted in the same way as explained for the 3 m^3^ reactor in Daub et al. [24]. All experiments were conducted with the same production batch of paraffin oil (Weissöl Ph Eur., Brenntag, Germany).

### Power input and power number Po

Power input was determined in different ways for the three reactors due to different technical limitations in the different scales. The 50L reactor was not equipped with power measurement. The power input was estimated using the equation from Middleton and Smith [54]:

(8) $$P=0.18\cdot Fl^{-0.2}\cdot Fr^{-0.25}\cdot P_{0}$$

with power input under aeration P, the impeller flow number Fl, impeller Froude number Fr and the unaerated power input P_0_ that can be calculated from

(9) $$P_{0}=\mathrm{Po}\cdot \rho_{c}\cdot n^{3}\cdot d^{5}$$

where Po is the power number, ρ_c_ the continuous phase density, n the agitation rate and d the impeller diameter. Power numbers were estimated using the equation of Liepe et al. [26]:

(10) $$\mathrm{Po}=5.9\cdot {\left(n_{\mathrm{bl}}^{0.8}\cdot \frac{h}{d}\right)}^{0.9}$$

where n_bl_ is the number of impeller blades and h is the impeller blade height, except for impeller configurations B-1 and B-3 where the power number was measured based on experiments without aeration. The reliability of this correlation can be tested by comparing the measured values for B-1 and B-3 with the calculated power numbers for these impeller configurations. The calculated power number for configuration B-1 is 5.5 vs. the measured value of 4.9 (+12%) and for configuration B-3 5.8 vs. the measured value of 5.9 (-2%). Both values are in reasonable agreement with the measured values. This correlation for power number can be considered very helpful and reliable within engineering accuracy and within the accuracy needed for the power data for the analyses conducted in this work. It resembles correctly the relative influence of blade height and impeller diameter.

Power input in the 3 m^3^ and in the 40 m^3^ reactors were measured through the electrical power draw of the engine corrected for friction and other losses. For the 3 m^3^ reactor the power input to the liquid was calculated from the raw value by a linear correction function as described in detail in Daub et al. [24]. Power losses were evaluated by an instationary temperature method that is completely independent of the electrical power measurement. The correlation for power losses was tested against electrical power measurement in the empty reactor and both measurements of power loss were in good agreement. Reproducibility of the electrical power measurement was very good with a standard deviation of 5%. For the 40 m^3^ reactor a linear correlation of the power data with n^3^ showed a good correlation of the data with an R^2^ of 0.99 when an offset of 25.2 kW was used. The two lowest agitation rates had very low power inputs (0.13 kW/m^3^ for n = 0.78 1/s and 0.36 kW/m^3^ for n = 1.0 1/s). It was decided to use calculated values for power input for these operating conditions instead of the measured values to avoid large measurement errors in this low range of operating conditions.

### Notation

a: Constant [-]

C: Bottom clearance of first impeller [m]

ΔC: Impeller spacing [m]

d: Impeller diameter [m]

d_max_: Maximum stable drop size [μm]

D_R_: Reactor diameter [m]

d_32_: Sauter mean diameter

Fl: Impeller flow number [-]

Fr: Impeller Froude number [-]

h: Impeller blade height [m]

H: Unaerated liquid height [m]

K_1_: Constant in Eq. 2 [-]

K_2_: Constant in Eq. 2 [-]

n: Agitation rate [1/s]

n_bl_: Number of impeller blades [-]

P: (Aerated) power input [W]

P_0_: Unaerated power input [W]

Po: Power number [-]

u_t_: Impeller tip speed [m/s]

V_L_: Total liquid volume [m^3^]

w: Impeller blade width [m]

#### Greek letters

ϵ_Ø_: Volume averaged energy dissipation rate [W/kg]

ϵ_max_: Maximum local energy dissipation rate [W/kg]

ρ_c_: Continuous phase density [kg/m^3^]

ϕ: ϵ_max_/ϵ_Ø_ [-]

σ: Interfacial tension between dispersed and continuous phase [N/m]

η_D_: Dynamic viscosity of dispersed phase [Pa∙s]

## Competing interests

The authors declare that they have no competing interests.

## Authors’ contributions

AD developed the method, performed experiments and prepared the manucript. SD, MB and MM performed experiments and contributed to experimental planning and analysis of data. GS and JB initiated the project. GS gave important guidance for the experimental work. JB contributed to method development, data analysis and manuscript preparation. All authors approved the final manuscript.
